# Supplementary material for: Integrated analysis of the aqueous humor microbiome and lens capsule transcriptome in high myopia cataract: a pilot study
Source: Front Med (Lausanne). 2026 Jun 16;13:1845205. doi: 10.3389/fmed.2026.1845205 (PMC13314463; doi:10.3389/fmed.2026.1845205)
Supplement: Supplementary file 3 [file Data_Sheet_1.zip › 2.Alpha_Diversity/alpha_boxplot/C372089/alpha_estimator_boxplot_shannon.pdf]

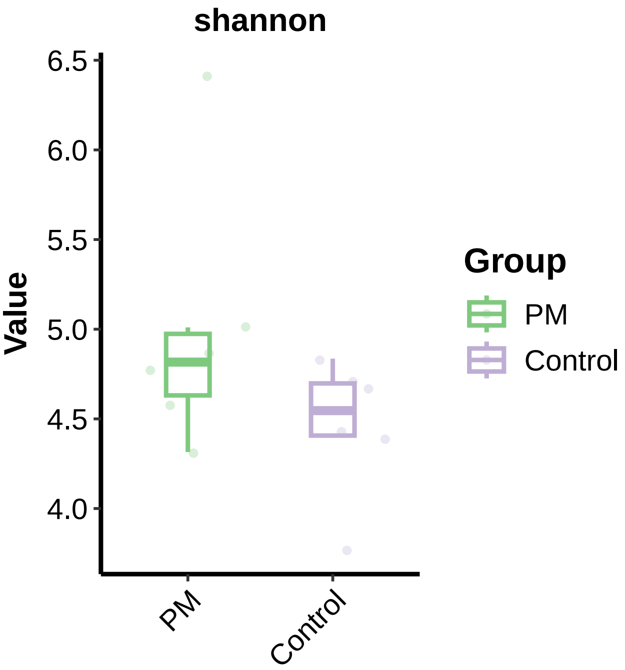
shannon
Value
6.5
6.0
5.5
5.0
4.5
4.0
PM
Control
Group
PM
Control
